# Rapid transformation of low-grade endometrial stromal sarcoma to *BCOR*-rearranged high-grade endometrial stromal sarcoma in a patient with Birt–Hogg–Dubé syndrome: A case report

**DOI:** 10.1016/j.gore.2026.102238

**Published:** 2026-10-21

**Authors:** Eko Okubo, Satoko Eguchi, Hiroko Tsuchiya, Reiko Matsuyama, Akihisa Fujimoto, Osamu Nishii, Tatsuya Aso, Mikiko Takahashi

**Affiliations:** a Department of Obstetrics and Gynecology, University Hospital, Mizonokuchi, Teikyo University School of Medicine, 5-1-1 Futako, Takatsu-ku, Kawasaki, Kanagawa, Japan; b Department of Pathology, University Hospital, Mizonokuchi, Teikyo University School of Medicine, 5-1-1 Futako, Takatsu-ku, Kawasaki, Kanagawa, Japan

**Keywords:** Endometrial stromal sarcoma, High-grade transformation, Birt–Hogg–Dubé syndrome, *FLCN*, *BCOR*, *MDM2*

## Abstract

[Background] Low-grade endometrial stromal sarcoma (LG-ESS) generally follows an indolent clinical course, and high-grade transformation during disease progression is rare. [Case Presentation] We report a 44-year-old woman with Birt–Hogg–Dubé (BHD) syndrome who developed rapid transformation of LG-ESS to high-grade endometrial stromal sarcoma (HG-ESS). The patient underwent surgery for a presumed uterine leiomyoma and was diagnosed with FIGO stage IA LG-ESS. Six months after surgery, a vaginal cuff recurrence was detected and rapidly progressed despite letrozole treatment. Cytoreductive surgery was performed, and the recurrent tumor was diagnosed as HG-ESS. *Re*-review of the primary specimen revealed no high-grade component. Comprehensive genomic profiling of the recurrent tumor identified a *ZC3H7B–BCOR* fusion and *MDM2* amplification, in addition to the germline pathogenic *FLCN* variant associated with BHD syndrome. Doxorubicin achieved a partial response, but the disease subsequently progressed during further treatment. This case demonstrates exceptionally rapid transformation from LG-ESS to *BCOR*-rearranged HG-ESS within 6 months. Although the temporal acquisition of the *BCOR* rearrangement could not be established because molecular testing was not performed on the primary tumor, the marked morphologic and clinical progression supports substantial tumor evolution. The identification of *BCOR* rearrangement and *MDM2* amplification may provide insight into the aggressive clinical course. [Conclusions] This case highlights the potential value of comprehensive genomic profiling in recurrent ESS and suggests that MDM2 inhibitors may represent a future treatment strategy for selected patients.

## Highlights

• Rapid transformation of low-grade endometrial stromal sarcoma in a 44-year-old woman.
• Transformation to *BCOR*-rearranged high-grade sarcoma occurred within 6 months.
• *BCOR* rearrangement and *MDM2* amplification were identified.
• Birt-Hogg-Dubé syndrome may have influenced rapid transformation to HG-ESS.

## 1 Introduction

Low-grade endometrial stromal sarcoma (LG-ESS) is a rare uterine mesenchymal neoplasm that typically affects premenopausal women and generally follows an indolent clinical course with a favorable prognosis. Advances in molecular pathology have led to a refined classification of endometrial stromal tumors, and the 2020 World Health Organization classification of female genital tumors recognizes LG-ESS and high-grade endometrial stromal sarcoma (HG-ESS) as distinct molecular entities. Yet there are rare reports of LG-ESS demonstrating histologic high-grade transformation (Zou et al., 2020; Dessources et al., 2022). Recent molecular studies suggest that the acquisition of genetic alterations may contribute to both tumor progression and the emergence of high-grade morphology. In particular, *BCOR*-related abnormalities have emerged as characteristic molecular findings in a subset of HG-ESS and have been associated with aggressive clinical behavior (Lin et al., 2020).

Birt–Hogg–Dubé (BHD) syndrome is a rare autosomal dominant hereditary disorder caused by pathogenic variants in the tumor suppressor gene *FLCN*. Patients with BHD syndrome characteristically develop pulmonary cysts, spontaneous pneumothorax, and fibrofolliculomas and are at increased risk for renal neoplasms (Tong et al., 2018). There are also proposed associations of BHD with several nonrenal malignancies, but the available evidence for these remains inconclusive; renal cancers are the only neoplasms with a well-established association. To date, there is no reported association between BHD syndrome and uterine sarcoma.

Herein, we report our experience with a patient with BHD syndrome who had an exceptionally rare clinical scenario of LG-ESS that underwent high-grade transformation to *BCOR*-rearranged HG-ESS at first recurrence, only 6 months after primary surgery.

## 2 Case presentation

A 44-year-old nulligravid premenopausal woman with a history of BHD syndrome was referred to our institution for surgical management of a presumed uterine leiomyoma associated with heavy menstrual bleeding. At 42 years of age, she had developed a spontaneous pneumothorax, which led to the identification of multiple bilateral pulmonary cystic lesions. She underwent thoracoscopic partial diaphragmatic resection, total pleural covering, and wedge resection of the lung. Subsequent genetic testing of her lung tissue revealed a pathogenic variant in *FLCN*, confirming the diagnosis of BHD syndrome. Her family history included gastric cancer in her father, paternal grandmother, 2 paternal uncles, and 1 maternal uncle, and breast cancer in her mother. No family members had a known diagnosis of BHD syndrome.

Transvaginal ultrasonography at her initial visit to our institution revealed an intramural mass measuring 4.5 cm in diameter in the posterior wall of the uterine corpus. Contrast-enhanced pelvic magnetic resonance imaging (MRI) demonstrated a heterogeneously hyperintense lesion on T2-weighted images. The imaging findings were considered consistent with a degenerating leiomyoma; no features suggestive of uterine sarcoma were identified (Fig. 1-a,b). The complete blood count, blood chemistry, coagulation tests, and tumor marker levels were within normal limits.

**Fig. 1:**
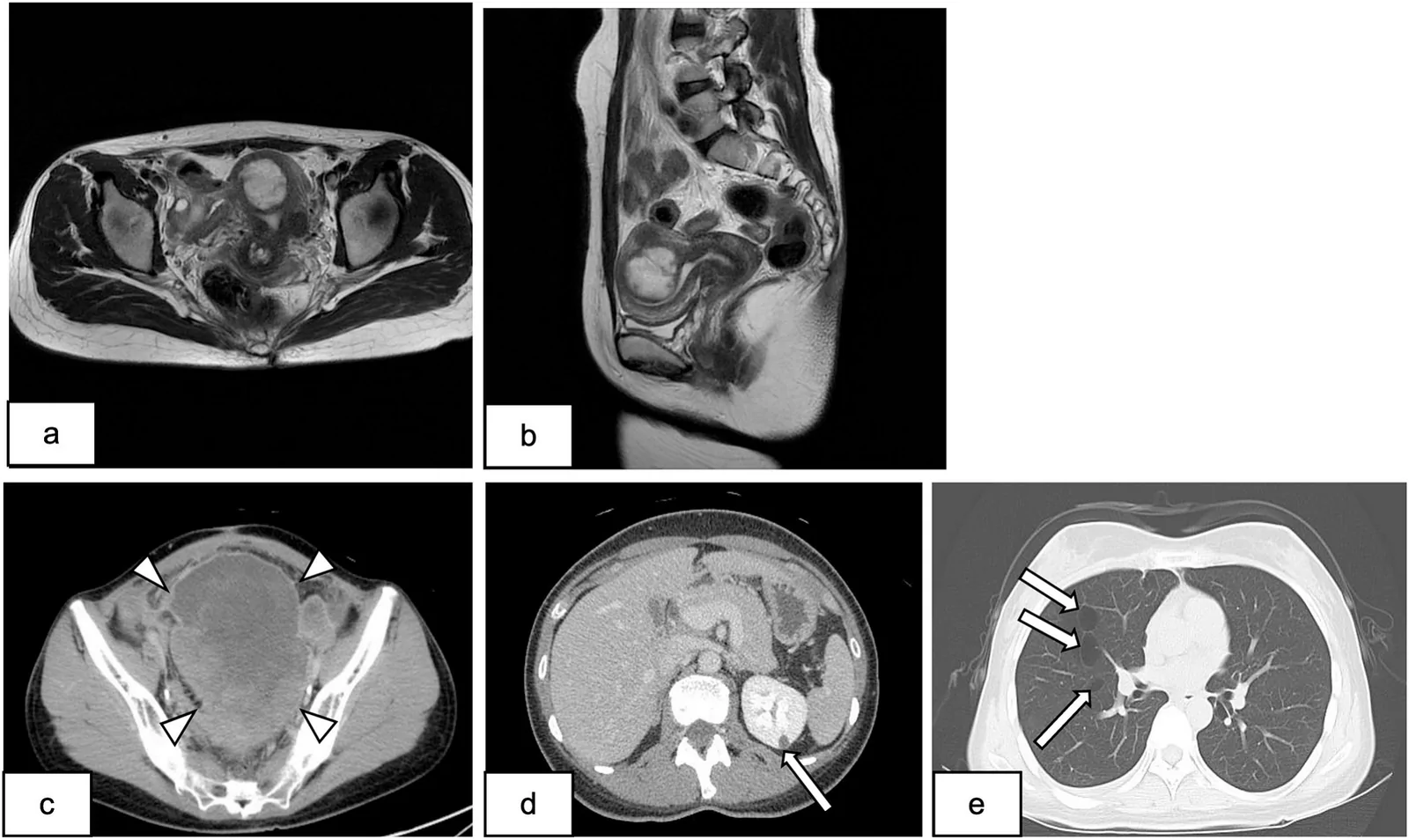
Pelvic magnetic resonance imaging (MRI) before the initial surgery (a,b) and Contrast-enhanced computed tomography (CT) 2 months after the detection of recurrence (c-e). a, b: Pelvic MRI showing a 4.5-cm intramural mass in the posterior uterine wall, suggestive of a degenerating leiomyoma, with no imaging features suggestive of uterine sarcoma. c: CT showing rapid enlargement of the pelvic mass to 14.5 cm in diameter (arrows). d, e: CT showing multiple pulmonary and renal cysts (arrowheads) consistent with BHD syndrome, with no distant metastases.

After 2 months of preoperative treatment with relugolix, the patient underwent laparoscopic myomectomy. The mass appeared to have degenerative changes and focally indistinct margins intraoperatively, but its gross appearance was consistent with a degenerating leiomyoma, and malignancy was not suspected. The tumor was retrieved in a retrieval bag through the umbilical incision. Histopathologic examination revealed a proliferation of enlarged round cells with mild cytologic atypia and high cellularity; mitotic figures and tumor necrosis were identified. Immunohistochemically, the tumor cells were positive for CD10, negative for estrogen receptors (ERs), and weakly positive for progesterone receptors (PgRs). Based on these findings, the tumor was diagnosed as LG-ESS (Fig. 2).

**Fig. 2:**
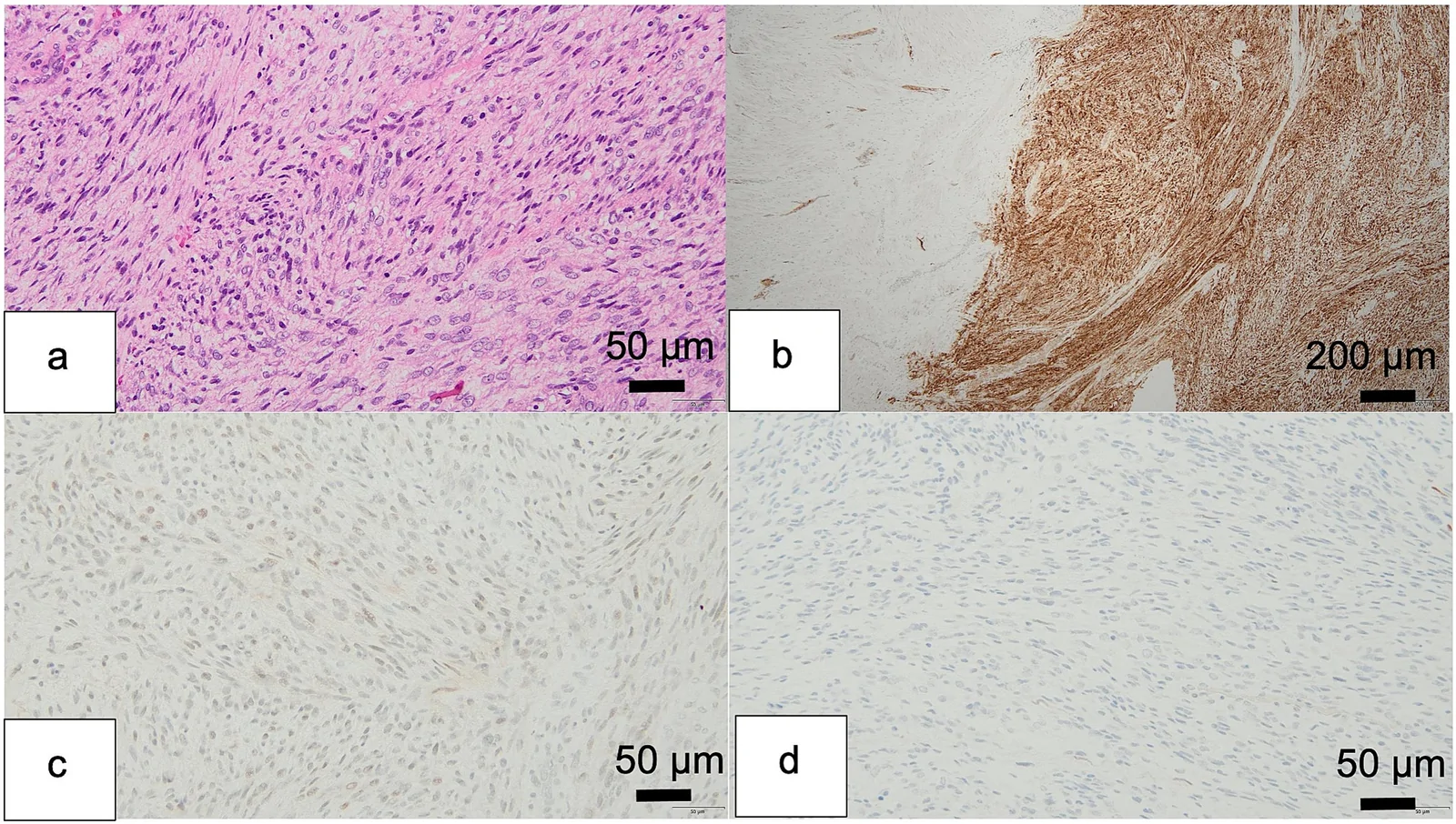
Histopathological findings of the tumor. a: Hematoxylin and eosin (H&E) staining showing a proliferation of enlarged round cells with high cellularity and mild cytologic atypia. b: Immunohistochemical staining showing positive expression of CD10. c: Immunohistochemical staining showing negative expression of estrogen receptor (ER). d: Immunohistochemical staining showing weakly positive expression of progesterone receptor (PgR).

Subsequently, the patient underwent a total abdominal hysterectomy, bilateral salpingo-oophorectomy, and peritoneal biopsy. No residual tumor was found in the resected uterus or adnexae. The final diagnosis was LG-ESS, FIGO 2018 stage IA. No adjuvant therapy was administered, and the patient was managed with surveillance.

Six months after surgery, a 6-cm mass was identified in the vaginal cuff, raising suspicion for recurrent LG-ESS. Treatment with letrozole (2.5 mg/day) was initiated, and surgical resection was planned. However, the tumor subsequently demonstrated rapid progression. Contrast-enhanced computed tomography (CT) performed 2 months later revealed rapid enlargement of the tumor to 14.5 cm in maximum diameter, and a 3-cm peritoneal implant was identified in the left external iliac region (Fig. 1-c). Multiple pulmonary and renal cysts consistent with BHD syndrome were also noted, but no distant metastases were detected (Fig. 1-d,e). Because of worsening abdominal pain, the patient underwent semi-urgent surgery. At the time of laparotomy, the vaginal cuff tumor had enlarged to approximately 20 cm in diameter, with partial capsular rupture and spillage of tumor contents into the abdominal cavity. Because the tumor was extensively adherent to the bladder, rectum, and pelvic peritoneum, precluding complete resection, maximal cytoreductive surgery was performed.

Histopathologic examination of the resected tumor revealed a densely cellular proliferation of enlarged round cells with an irregular growth pattern and moderate cytologic atypia; frequent mitotic figures were observed. Immunohistochemically, the tumor cells were ER and PgR negative, whereas the primary tumor had shown weak PgR positivity. The Ki-67 labeling index was markedly elevated, at 80%. Based on these findings, the tumor was diagnosed as HG-ESS (Fig. 3). *Re*-evaluation of the initial surgical specimen revealed no histologic evidence of a high-grade component. Therefore, the recurrent tumor was considered to represent high-grade transformation of the original LG-ESS. Doxorubicin monotherapy (75 mg/m^2^) was initiated 2 weeks after surgery. A partial response was confirmed on CT, and treatment was continued for 6 cycles.

**Fig. 3:**
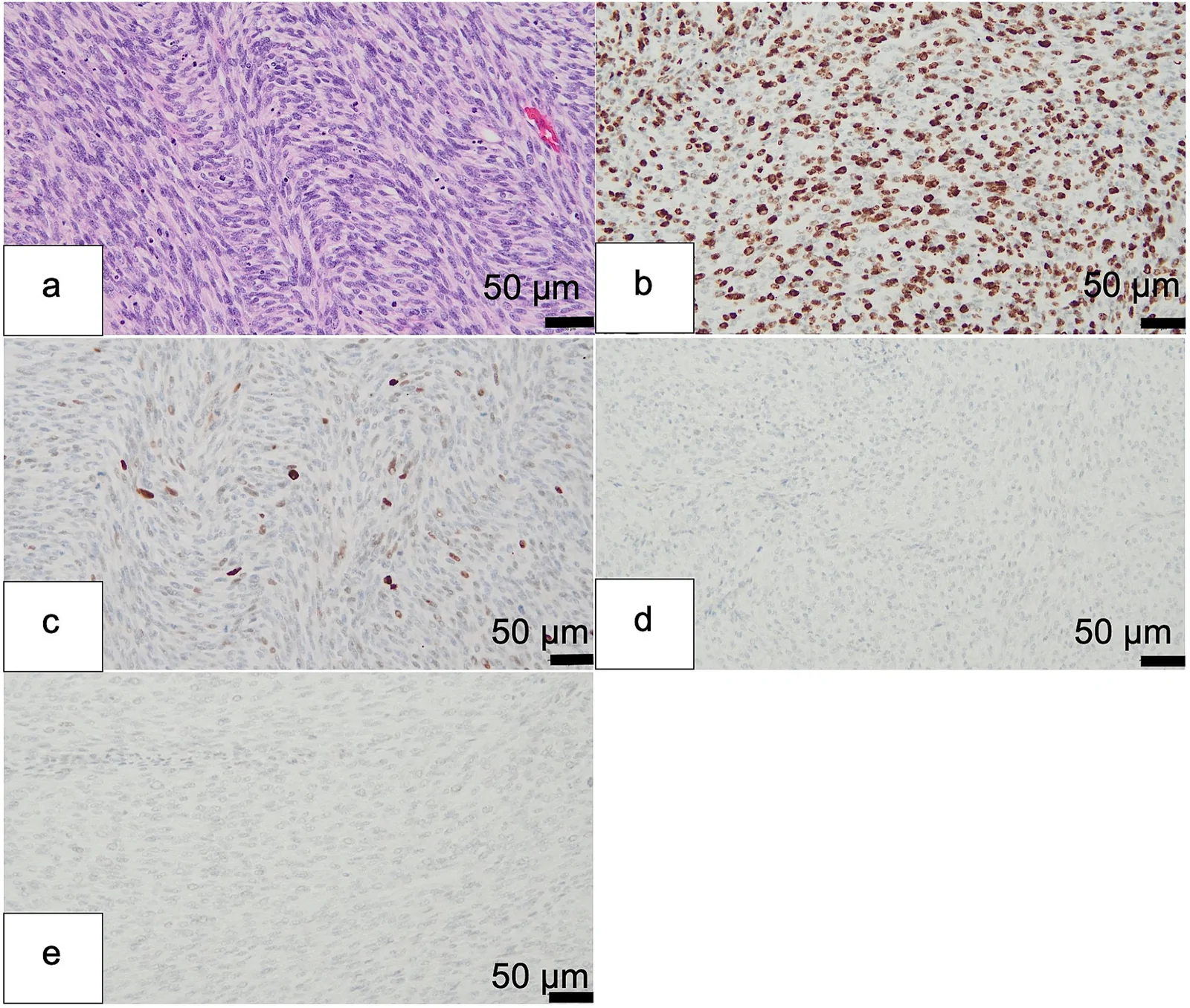
Histopathological findings of the recurrent tumor. a: Hematoxylin and eosin (H&E) staining showing a densely cellular proliferation of enlarged round cells with moderate cytologic atypia, an irregular growth pattern, and frequent mitotic figures. b: Immunohistochemical staining showing a high Ki-67 labeling index (80%). c: Immunohistochemical staining for p53 showing a wild-type expression pattern. d: Immunohistochemical staining showing negative expression of estrogen receptor (ER). e: Immunohistochemical staining showing negative expression of progesterone receptor (PgR), in contrast to weak positivity in the primary tumor.

Comprehensive genomic profiling (GenMineTOP; Tokyo, Japan) of the recurrent tumor identified a *ZC3H7B*–*BCOR* fusion gene, *MDM2* amplification (55.1 copies), and the germline pathogenic *FLCN* variant that had originally been identified. The tumor mutational burden was low (0.5 mutations/Mb), and the tumor was microsatellite stable. Although treatment with an *MDM2* inhibitor was proposed as a potential therapeutic option, there were no current clinical trials for which the patient was eligible. Therefore, systemic treatment with approved therapies was recommended.

Immediately after completion of 6 cycles of doxorubicin, rapid tumor regrowth was noted. Because of small bowel invasion, an ileostomy was performed, followed by initiation of eribulin therapy (1.1 mg/m^2^). However, the disease continued to progress, accompanied by deterioration of the patient's general condition. The patient was transitioned to best supportive care 18 months after the initial surgery.

## 3 Discussion

LG-ESS is generally characterized by an indolent clinical course, prolonged survival, and a tendency for late recurrence. Although recurrent disease is relatively common, the development of high-grade morphology during disease progression is considered rare. Recent studies have documented patients with LG-ESS exhibiting high-grade histologic transformation, suggesting that the acquisition of genetic alterations may contribute to tumor progression in a subset of patients (Zou et al., 2020; Lin et al., 2020). *BCOR* alterations have emerged as characteristic molecular findings in a subset of high-grade endometrial stromal sarcomas and are associated with aggressive clinicopathologic features. These observations raise the possibility that *BCOR*-related abnormalities may contribute to the acquisition of high-grade morphology during tumor progression. However, the molecular mechanisms underlying this process remain incompletely understood. In our patient, the identification of a *BCOR* rearrangement in her recurrent high-grade tumor provides further insight into the potential role of *BCOR*-related alterations in disease progression.

The most remarkable feature in our patient was the extremely short interval between her initial treatment of LG-ESS and her development of HG-ESS. Previous reports describing high-grade transformation of LG-ESS have generally documented recurrence several years after the initial diagnosis, with some patients developing transformed disease more than a decade after primary treatment (Zou et al., 2020; Dessources et al., 2022). In contrast, our patient developed recurrent disease with histologically confirmed HG-ESS only 6 months after primary surgery. *Re*-review of the primary tumor confirmed the absence of a high-grade component, supporting the interpretation that the recurrent lesion represented high-grade transformation rather than an initially unrecognized HG-ESS. To the best of our knowledge, such transformation within 6 months of primary treatment has rarely been reported.

Comprehensive genomic profiling of our patient's recurrent tumor identified *BCOR* rearrangement and *MDM2* amplification. *BCOR* rearrangements are recognized as characteristic molecular alterations in a subset of HG-ESS and are thought to contribute to tumor progression and acquisition of high-grade morphology (Lin et al., 2020; Kommoss et al., 2020; Astolfi et al., 2019). The detection of *BCOR* rearrangement in our patient is therefore consistent with the diagnosis of HG-ESS and supports the concept that the recurrent tumor had undergone genuine biological transformation. Nevertheless, because molecular testing was not performed on the primary tumor, the temporal relationship between *BCOR* rearrangement and high-grade transformation could not be definitively established. It therefore remains uncertain whether the *BCOR* rearrangement was acquired during disease progression or was already present in a minor subclone of the primary tumor. However, the absence of a high-grade component in the extensively reviewed primary specimen, together with the marked morphologic and clinical progression at recurrence, supports the interpretation that substantial tumor evolution occurred during the interval.

In addition to *BCOR* rearrangement, the recurrent tumor demonstrated *MDM2* amplification. As a key negative regulator of p53, *MDM2* amplification may facilitate tumor progression through attenuation of p53-mediated tumor suppressive activity (Wade et al., 2013). Recent studies have shown that *MDM2* amplification is relatively frequent in *BCOR*-rearranged HG-ESS, suggesting a potential biological relationship between these alterations (Kommoss et al., 2020). Although its contribution to high-grade transformation remains unclear, *MDM2* amplification may have contributed to the unusually aggressive clinical course observed in our patient.

Another unique aspect of this case was the presence of underlying BHD syndrome. BHD syndrome is an autosomal dominant disorder caused by pathogenic variants in *FLCN* and is classically associated with pulmonary cysts, spontaneous pneumothorax, renal neoplasms, and characteristic skin lesions. To our knowledge, an association between BHD syndrome and HG-ESS has not previously been reported. Although *FLCN* is involved in mTOR-related signaling and cellular metabolic regulation, the mechanisms linking *FLCN* deficiency to malignant progression remain incompletely understood (Ramirez Reyes et al., 2021; Schmidt and Linehan, 2017). Nevertheless, disruption of *FLCN*-related growth-regulatory pathways may have contributed to the unusually aggressive clinical course observed in this patient. While a causal relationship cannot be established from a single case, the coexistence of BHD syndrome raises the possibility that background genetic factors may influence the progression of uterine stromal neoplasms.

Several pathologic features observed at the time of this patient's initial diagnosis may also have predicted an increased risk of aggressive tumor behavior. Previous studies have suggested that p53 abnormalities are associated with aggressive neoplastic characteristics and may precede high-grade transformation, whereas loss of hormone-receptor expression has been associated with an increased risk of recurrence (Ohta et al., 2010; Huang and Peng, 2022). Although p53 overexpression was not observed in our patient's primary tumor, it was ER negative and demonstrated low PgR expression; the latter was not present in the recurrent tumor. These findings may suggest the presence of biological features associated with an increased risk of aggressive behavior.

The genomic findings also have potential therapeutic implications. *MDM2* amplification has emerged as a potential treatment target in several sarcoma subtypes, and recent studies have demonstrated preliminary antitumor activity of MDM2 inhibitors in *MDM2*-amplified tumors (Koyama et al., 2023; Gounder et al., 2023). Although no clinical evidence currently supports their use in patients with HG-ESS, the identification of *MDM2* amplification in our patient's tumor suggests that such agents may represent a rational therapeutic strategy for selected patients. Unfortunately, no suitable clinical trials were available for our patient. In contrast, mTOR inhibitors were not suggested for our patient by genomic profiling, despite the presence of BHD syndrome. Given the complex relationship between *FLCN* dysfunction and mTOR signaling, and the limited evidence supporting mTOR inhibition in BHD-associated tumors, *MDM2* amplification represented a more direct and actionable therapeutic target for our patient.

## 4 Conclusion

We report our experience with a patient who had BHD syndrome and an exceptionally rare histologically confirmed transformation from LG-ESS to *BCOR*-rearranged HG-ESS within 6 months of primary surgery. The identification of *BCOR* rearrangement and *MDM2* amplification in her tumor suggests that these molecular alterations may have contributed to the unusually aggressive clinical course. In addition, her underlying BHD syndrome and FLCN dysfunction may have acted as disease-modifying factors, although a causal relationship remains speculative.

This case highlights the potential value of comprehensive genomic profiling in recurrent ESS and raises the possibility that MDM2-directed therapies may represent a future treatment strategy for selected patients with HG-ESS. Further accumulation of case reports and molecular investigations will be required to clarify the mechanisms responsible for rapid high-grade transformation and to identify clinically actionable therapeutic targets.

## 5 Consent

Written informed consent was obtained from the patient for publication of this case report and accompanying images.

## CRediT authorship contribution statement

**Eko Okubo:** Writing – review & editing, Writing – original draft, Investigation. **Satoko Eguchi:** Writing – review & editing, Writing – original draft, Supervision, Investigation, Conceptualization. **Hiroko Tsuchiya:** Writing – review & editing. **Reiko Matsuyama:** Writing – review & editing. **Akihisa Fujimoto:** Writing – review & editing. **Osamu Nishii:** Writing – review & editing. **Tatsuya Aso:** Writing – review & editing, Investigation. **Mikiko Takahashi:** Writing – review & editing, Investigation.

## Ethics declaration

### Informed consent and patient details

Written informed consent to take part in the study and to publish the article has been obtained from all participants or their legal representatives. The privacy rights of participants have been observed.

### Human and biological material

This study included organ or tissue donors.

This study includes human biological material and consent was obtained by donors, or their next of kin or legal representatives, for use in this study and for publication of the article. The samples used in this research were not sourced from executed prisoners or prisoners of conscience.

Studies in human

This study was performed in compliance with relevant laws, regulatory frameworks and guidelines where the research took place.

Ethics committee approval was not required under relevant laws and institutional guidelines. Ethical approval was not required for this case report in accordance with institutional guidelines. Written informed consent for publication of the case report and accompanying clinical information was obtained from the patient.

### Case reports

This research follows the CARE guidelines and the CARE checklist.

## Declaration of competing interest

The authors declare that they have no known competing financial interests or personal relationships that could have appeared to influence the work reported in this paper.
